# Metabolite profiling at the cellular and subcellular level reveals metabolites associated with salinity tolerance in sugar beet

**DOI:** 10.1093/jxb/erx388

**Published:** 2017-11-11

**Authors:** M Sazzad Hossain, Marcus Persicke, Abdelaleim Ismail ElSayed, Jörn Kalinowski, Karl-Josef Dietz

**Affiliations:** 1 Department of Biochemistry and Physiology of Plants, Faculty of Biology, Bielefeld University, Universitätsstr.25, Germany; 2 Center for Biotechnology-CeBiTec, Bielefeld University, Universitätsstr. Germany; 3 Biochemistry Department, Faculty of Agriculture, Zagazig University, Egypt

**Keywords:** Chloroplast, metabolite profiling, non-aqueous fractionation, photosynthesis, salinity stress, sugar beet

## Abstract

Sugar beet is among the most salt-tolerant crops. This study aimed to investigate the metabolic adaptation of sugar beet to salt stress at the cellular and subcellular levels. Seedlings were grown hydroponically and subjected to stepwise increases in salt stress up to 300 mM NaCl. Highly enriched fractions of chloroplasts were obtained by non-aqueous fractionation using organic solvents. Total leaf metabolites and metabolites in chloroplasts were profiled at 3 h and 14 d after reaching the maximum salinity stress of 300 mM NaCl. Metabolite profiling by gas chromatography-mass spectrometry (GC-MS) resulted in the identification of a total of 83 metabolites in leaves and chloroplasts under control and stress conditions. There was a lower abundance of Calvin cycle metabolites under salinity whereas there was a higher abundance of oxidative pentose phosphate cycle metabolites such as 6-phosphogluconate. Accumulation of ribose-5-phosphate and ribulose-5-phosphate coincided with limitation of carbon fixation by ribulose-1,5-bisphosphate carboxylase/oxygenase (Rubisco). Increases in glycolate and serine levels indicated that photorespiratory metabolism was stimulated in salt-stressed sugar beet. Compatible solutes such as proline, mannitol, and putrescine accumulated mostly outside the chloroplasts. Within the chloroplast, putrescine had the highest relative level and probably assisted in the acclimation of sugar beet to high salinity stress. The results provide new information on the contribution of chloroplasts and the extra-chloroplast space to salinity tolerance via metabolic adjustment in sugar beet.

## Teaser

The use of non-aqueous fractionation of leaf samples with organic solvents allows differences in subcellular localization of metabolites to be determined in response to salinity stress in sugar beet.

## Introduction

Sugar beet (*Beta vulgaris* L.) is considered to be a crop that is highly tolerant to drought and salt stress (Hajheidari *et al.*, 2005; Li *et al.*, 2009; Yang *et al.*, 2012; Wedeking *et al.*, 2017). It shows a high ability for osmotic adjustment through the accumulation of inorganic ions such as sodium, potassium, and chloride in leaves in response to salinity and/or drought (Ghoulam *et al.*, 2002). In many species growth in saline soil leads to dehydration, ionic stress, nutritional deficiencies, and oxidative stress, with the main negative effects being the disruption of ionic equilibrium and the inhibition of cell division and expansion (Heidari *et al.*, 2011). Expression of salinity tolerance depends on often conditional activation of complex physiological traits and metabolic pathways that are triggered through a sensory signaling network (Sanchez *et al.*,2008, 2011). Upon exposure to salinity, plants accumulate a range of osmolytes for osmotic adjustment, alter their metabolism to stabilize proteins and cellular structures, and control metabolism to remove excess reactive oxygen species (ROS) in order to re-establish the cellular redox balance (Bartels and Sunkar, 2005; Valliyodan and Nguyen, 2006; Munns and Tester, 2008; Janska *et al.*, 2010). Important plant metabolites that have been implicated in acclimation to salinity include sugars, amino acids, polyols, dimethylsulfonium, and polyamines. They serve as osmoprotectants and osmolytes, and protect plant cells under environmental stresses such as salt, drought, frost, and heat (Shulaev *et al.*, 2008; Nazar *et al.*, 2011). Plants have a remarkable ability to synthesize a vast array of substances categorized as primary metabolites, including sugars, amino acids, nucleotides, and lipids, that are implicated in essential life functions such as nutrition, growth, and reproduction. On the other hand, secondary metabolites often affect ecological interactions between the plant and its environment (Croteau *et al.*, 2000). Secondary metabolites produce beneficial effects, for example, they protect membranes and increase activities of antioxidant enzymes that in turn scavenge ROS and counteract lipid peroxidation (Baque *et al.*, 2012). They contribute to the overall fitness of plants under adverse growth conditions (van der Fits and Memelink, 2000).

Metabolomics studies play a central role in the post-genomic era since they allow the characterization of physiological responses to different types of environmental stresses in plants (Kaplan *et al.*, 2004). Gas chromatography-mass spectrometry (GC-MS) is still the most widely used technique for the separation, identification, and quantification of metabolites such as amino acids, sugars, sugar alcohols, polyamines, and organic acids (Schauer and Fernie, 2006). For more comprehensive metabolite profiling, samples nowadays are often analysed both by LC-MS and GC-MS (Kopka *et al.*, 2004).

Our previous work has revealed the efficiency of sugar beet in regulating its cellular redox homeostasis and antioxidant defense under high salinity despite massive accumulation of sodium and chloride (Hossain *et al.*, 2017). Compartmentation is a fundamental process in salt tolerance (Yeo, 1998). Combining metabolite profiling with an examination of subcellular compartmentation enables a broad assessment of metabolic alterations and allows us to address the question of the underlying metabolic alterations that might contribute to the particular redox response seen in sugar beet. However, concentrations of metabolites are usually analysed in whole tissues and hence only provide a general picture of alterations; such information misses the level of compartmentation. Compartmentation of pathways and activities is a fundamental property of life and several mechanisms for its occurrence were already in place in prokaryotes, e.g. by super-complex formation or membrane association, but it is particularly elaborated in eukaryotes including plants. In mesophyll cells of spinach and barley the chloroplast occupies about 25–30% of the total volume (Winter *et al.*, 1993, 1994) representing the largest plasmatic compartment. The leaves of spinach and sugar beet have a highly similar morphology, and thus data derived from spinach can probably be applied reasonably well to sugar beet. An important gap exists in most metabolite studies concerning the temporal and, in particular, the spatial resolution of the *in vivo* state of the metabolites. Metabolic networks are highly dynamic and metabolites are specifically compartmentalized in subcellular compartments. Therefore, studying subcellular metabolites is important to achieving a deeper understanding of how plants respond to abiotic stresses. It is possible to determine the subcellular metabolite contents of leaves *ex vivo*. Non-aqueous fractionation of leaves was introduced 60 years ago by Heber (1957), who separated a chloroplast fraction from the residual material of the leaf tissue. As the site of photosynthesis and specific metabolism, the chloroplasts play a fundamental role in stress acclimation. The mesophytic characteristics of sugar beet leaves make them well suited for non-aqueous isolation of chloroplasts (Dietz, 1985). The non-aqueous fractionation technique was modified by Gerhardt and Heldt (1984) to address metabolite concentrations in other subcellular fractions including the vacuole and mitochondria. This method was used, for example, by Krueger *et al.* (2011) to produce a subcellular map of metabolites in Arabidopsis leaves. The method suffers from several drawbacks, in particular the fact that leaves are homogenized prior to separation and this makes it impossible to ascertain whether differences exist between individual cell types (e.g. mesophyll versus non-mesophyll cells). In addition, the method relies on identification of marker enzymes associated with particular individual subcellular compartments against which metabolites can be measured. However, these associations may not be precise; for example, α-mannosidase is associated with the vacuole and also with the apoplast and cell wall and other endomembrane compartments (Martinoia *et al.*, 1981; Betz *et al.*, 1992).

The role of chloroplast metabolism under extreme salinity is important if we want to understand salinity acclimation. Sugar beet appears to be a particularly promising plant to elucidate the subcellular metabolite changes because of its high salinity tolerance and its value as a crop. The known effects of salinity on its ROS metabolism prompted us to address the role of related metabolic pathways with a particular focus on chloroplasts, which are a major source of ROS under stress (Hossain and Dietz, 2016). Chloroplast metabolism plays an important role under stress including salinity (Kmiecik *et al.*, 2016; Mignolet-Spruyt *et al.*, 2016; Bose *et al.*, 2017).

Determining the role of photosynthesis and subcellular localization is therefore an important step towards an understanding of cellular stress responses. Non-aqueous fractionation of chloroplasts allows the enrichment of a specific subcellular compartment derived from lyophilized material and essentially determines the *in vivo* state of metabolism. Rapid quenching of metabolism at –196 °C and lyophilizing of the tissue suppresses the enzymatic interconversion of metabolites. Initially, this method was applied to the separation and purification of chloroplasts by several centrifugation steps (Heber, 1957; Stocking, 1959). Non-aqueous fractionation is one of the most promising approaches for studying metabolite compartmentalization. The resulting metabolite composition should provide insights into the mechanisms of salt tolerance in sugar beet. Our objective was therefore to investigate the metabolic adaptations of sugar beet to salt stress through GC-MS analysis of whole leaf tissues and chloroplasts separated by non-aqueous fractionation.

## Materials and methods

### Plant material and salt stress treatments

Sugar beet seeds (cultivar KWS2320) were sterilized with 70% (v/v) ethanol, 0.1% (w/w) mercurial chloride, and 0.2% (w/w) thiram, then placed in a mix of vermiculite and perlite for germination, and soaked with water and maintained in the dark for 7 d. After germination the seedlings were maintained in the light for an additional week. When they were 14 d old, seedlings with uniform growth were transferred to hydroponic containers with Hoagland solution (Ghoulam *et al.*, 2002). Growth conditions were 10 h light (100 µmol m^–2^ s^–1^) at 21 °C and 14 h darkness at 18 °C with 55% relative humidity. Stressed plants were subjected to increasing concentrations of salt (NaCl) applied in 50-mM increments each day over 6 d until a final level of 300 mM was reached, as described previously (Hossain *et al.*, 2017) (see Supplementary Fig. S1 at *JXB* online). Both the control and salt-treated samples were harvested at 3 h and at 14 d after reaching the highest salinity level, at the same time of day. These time points were selected based on the results from a detailed time course investigated by Hossain *et al.* (2017) and also shown in Supplementary Fig. S1. The treated and control leaves were immediately frozen in liquid nitrogen in the light and subsequently freeze-dried at –40 °C. The material was stored at –80 °C in the presence of a strong desiccant in a closed plastic container Six independent experiments were conducted.

### Determination of CO_2_ fixation and quantum yield of photosystem II

CO_2_ fixation and the quantum yield of photosystem II (ΦPSII) of sugar beet leaves under control and salt-stress conditions were measured with a portable gas exchange fluorescence system (GFS-3000, Heinz Walz GmbH, Effeltrich, Germany) (Farooq *et al.*, 2016). The CO_2_ assimilation rate was measured at a light intensity of 100 μmol photons m^−2^ s^−1^, a relative humidity of 50% and at 22 °C (Oelze *et al.*, 2012). Measurements began at the time when the salt stress was increased to its maximum level of 300 mM NaCl.

### Determination of ribulose 1,5 bisphosphate carboxylase/oxygenase (Rubisco)

Rubisco activity was assessed according to Parry *et al.* (2002), with a few modifications, by coupling its activity to NADH oxidation using phosphoglycerate kinase and glyceraldehyde-3-phosphate dehydrogenase. Leaf samples (500 mg) were homogenized with 1 ml extraction buffer (0.1 M Tris pH 7.8, 5 mM MgCl_2_, 5 mM DTT, 0.1 mM EDTA, 1.5% polyvinylpyrrolidone) and centrifuged at 16 000 *g* for 10 min at 4 °C. The supernatant was used for determining the initial and total activity of Rubisco. The oxidation of NADH was measured at 340 nm using a spectrophotometer (Cary 300 Bio UV/VIS, Varian, Middelburg, Netherlands). Activity was calculated using a molar extinction coefficient of 6230 M^–1^ cm^–1^.

### Homogenization of the sample for chloroplast isolation by non-aqueous fractionation

A suitable homogenization knife was mounted in a Buehler homogenizer (Homogenizer HO 4/A, Edmund Bühler GmbH, Germany). The 50-ml Buehler flask was filled with about 1 g of freeze-dried leaf material, and hexane was added to half the height of the flask. The sample was homogenized five times for 30 s with a 1-min pause each time, and the flask was cooled during homogenization to prevent heating of the sample. The homogenate was filtered through two layers of muslin cloth and transferred to 50-ml centrifugation tube. Aliquots were then used for further analysis.

### Fractionation of the chloroplasts through gradient centrifugation

Aliquots from the step described above were centrifuged at 2000 rpm for 3 min at 4 °C (800 *g*; Heraeus™ Megafuge™, Thermo Fisher Scientific Inc.). Three-quarters of the volume of the supernatant was discarded and the sediment was re-suspended in the remaining supernatant. For the gradient centrifugation, a test series of density gradients was prepared from tetrachloroethylene (TCE; density 1.62 g cm^–3^) and petroleum ether (PET; density 0.673 g cm^–3^). The suspension was under-layered with mixtures of heavy (70–84% TCE/30–16% PET) and light (33% TCE/67% PET) solvent. The gradients were centrifuged at 2500 rpm (1000 *g*) for 5 min at 4 °C. The crude chloroplast fractions were collected from the interface (see Supplementary Fig. S2). For control plants, 76% TCE/24% PET with 33% TCE/67% showed the best chloroplast yield. The gradient was adjusted for the salt-stressed sample (78% TCE/22% PET, 33% TCE/67%). For purification, the chloroplast fraction was subjected to differential sedimentation using 33% TCE/67% PET. The first sedimentation was obtained at 1800 rpm (720 *g*) and 4 °C for 45 s. The supernatant containing lighter material was discarded. The sediment was resuspended and centrifuged at 1000 rpm (400 *g*) for 10 s at 4 °C. The supernatant was sedimented at 2500 rpm (1000 *g*) for 2 min at 4 °C to obtain purified chloroplasts, which were dried and transferred to new tubes. The dry chloroplasts were stored in a closed plastic vessel with a strong desiccant (Orange Silica Gel, Merck, Darmstadt) at –80 °C.

### Determination of chloroplast markers

#### Chlorophyll and protein contents

Chlorophyll was assayed according to Arnon (1949) and calculated as described by Porra (2002). Pulverized leaves (about 2 mg) or isolated chloroplasts (about 0.5 mg) were extracted in 1 ml of acetone:H_2_O (4:1 v/v) with gentle agitation in the dark for 24 h at 4 °C. The extracts were centrifuged, and absorbance was measured using a spectrophotometer (Cary 300 Bio UV/VIS, Varian, Middelburg, Netherlands). Protein content was determined by the Bradford method with bovine serum albumin as a standard (Bradford, 1976).

#### Determination of enzyme activities

Enzyme extracts were prepared by addition of 0.5 ml of 0.25 mM K-phosphate, pH 7.5, containing 0.5 mM DTT and 0.5 mM EDTA to the dried samples (10 mg). The tubes were shaken three times for 30 s, with a pause of 1 min each time, using a cell disintegrator on ice. Afterwards, a buffer containing 0.5 ml 100 mM K-phosphate, pH 7.5, 0.5 mM DTT, and 0.5 mM EDTA was added and the clear supernatant was used to assay enzyme activity.

NADP-glyceraldehyde 3-P dehydrogenase activity was determined as a marker for the chloroplast (Cerff and Quail, 1974). The reaction mixture contained 10 µl of extract and 40 mM Tris-HCL buffer (pH 7.8), 8 mM MgSO_4_, 2 mM dithioerythritol (DTE), 4.12 mM cysteine, 1.04 mM glutathione, 1.1 mM ATP, and 9 mM glycerate-3-P. After mixing and incubation at 25 °C for 10 min, the oxidation of NADPH was recorded at 366 nm (ɛ=3500 l mol^–1^ cm^–1^) and 25 °C. After recording the baseline absorbance for around 2 min, the reaction was started by adding 0.16 mM NADPH. Buffer was added to a blank control.

Phosphoenolpyruvate carboxylase (PEPC) activity was determined as a marker for the cytosol according to Price and Donkin (1982). Each cuvette contained 300 µl of extract and 150 µmol Tris-HCL, pH 7.8, 15 µmol MgCl_2_, 0.3 µmol EDTA, 7.5 µmol DTT (dithiothreitol), 15 µmol NaHCO_3_, 0.25 µmol NADH, 6 µmol phosphoenolpyruvate (PEP), and 4.2 U malate dehydrogenase. The reaction was started by adding PEP to the reaction cuvette, and distilled water to the blank control. Absorbance was monitored at 340 nm (ɛ=6.22 × 10^3^ l mol^–1^ cm^–1^) and 25 °C.

α-Mannosidase is considered as a marker for the endomembrane compartments including the vacuole and the apoplast (Martinoia *et al.*, 1981; Betz *et al.*, 1992). The enzyme activity was assayed according to the method of Gerhardt and Heldt (1984), which is based on the formation of *p*-nitrophenol from *p*-nitrophenyl α-D-mannoside. The enzyme activity was measured by adding 20 µl sample to 1 ml reaction medium that contained 50 mM citrate buffer, pH 4.5, and 5 mM *p*-nitrophenyl-α-D-mannoside. After incubation for 60 min at 37 °C, the reaction was stopped by adding 0.5 ml of 0.8 M borate buffer (pH 9.8), and the absorption was measured at 405 nm (ɛ=18500 l mol^–1^ cm^–1^) and 25 °C against individual blanks.

### Gas chromatography-mass spectrometry (GC-MS) analysis

#### Metabolite extraction from sugar beet leaf

A pulverized leaf sample (5 mg) was homogenized using a ribolyzer (3 × 45 s, 6.5 ms^–1^; Peqlab) with 0.5 g of silica beads (0.5 mm diameter; Roth) and 1 ml 80% methanol containing 10 µM ribitol as internal standard. A 600-µl aliquot of supernatant was dried in a stream of nitrogen gas and derivatized at 37 °C by addition of 75 µl methoxylamin-hydrochloride (20 mg ml^–1^ in pyridine; Sigma Aldrich) for 90 min and 75 µl N-methyl-N-(trimethylsilyl) trifluoroacetamide (Macherey–Nagel) for 30 min.

#### Peak detection of extracted metabolites

Analysis was performed on a GC–MS TRACE GC coupled to a Polaris *Q* mass spectrometer (Thermo Fisher Scientific) using the protocol of Plassmeier *et al.* (2007). The instrument was equipped with a Rtx®-5MS column (30 m, iD 0.25, df 0.25 µm; Restek). A 1-µl sample was injected in splitless mode into the GC-MS column. The oven program consisted of 3 min at 80 °C, a ramp with 5 °C min^–1^ up to 325 °C, and finally 325 °C for 2 min. The MS transfer line temperature to the quadruple was set to 250 °C and the electron impact (EI) ion source temperature was 220 °C. The spectra were recorded with a scanning range of 50–650 mz^–1^ (Supplementary Table S1). Replicate samples were derivatized and measured separately with intervals of at least 3 d. A blank was run every four samples to check for carry-over of metabolites.

#### Data analysis

Samples from six individual experiments were measured with at least three technical repeats. Peak detection in chromatograms was done with a signal-to-noise ratio of 5 followed by a multiple profiling to identify common MS patterns. Compounds and metabolites were identified according to the retention index (Kind *et al.*, 2009) and additional fitting of mass spectra to separately measured reference substances (Plassmeier *et al.*, 2007). Quantification was performed on peak areas of characteristic compound masses, normalized to ribitol (at 217 mz^–1^) and to the dry weight of the material, and detailed MS analysis was carried out using the Xcalibur 2.0.7 software (Thermo Fisher Scientific). All further processing of data was carried out using the ClustVis (http://biit.cs.ut.ee/clustvis/) software (Metsalu and Vilo, 2015).

#### Calculation of metabolite levels in compartments

Chloroplast and extra-chloroplast contents were calculated using a set of equations as outlined below. Input parameters were as follows (r.u. indicates relative units).

(1)  Metabolite level in non-aqueous total leaf (from GC-MS): *M*_T_ (r.u. mg^–1^ DW)
(2)  Metabolite level in non-aqueous chloroplast fraction (from GC-MS): *M*_Cf_ (r.u. mg^–1^ DW)
(3)  Chloroplast (P) enzyme marker activity in non-aqueous total leaf: *EP*_T_ (µmol mg^–1^ s^–1^)
(4)  Chloroplast enzyme marker activity in non-aqueous chloroplast fraction: *EP*_Cf_ [µmol mg^–1^ DW]
(5)  Cytosol (C) enzyme marker activity in non-aqueous total leaf: *EC*_T_ (nmol mg^–1^ s^–1^)
(6)  Cytosol enzyme marker activity in non-aqueous chloroplast fraction: *EC*_Cf_ (µmol mg^–1^ DW)
(7)  Chlorophyll content in non-aqueous total leaf: *Y*_T_ (µg mg^–1^ DW)
(8)  Chlorophyll content in non-aqueous chloroplast fraction: *Y*_Cf_ (µgmg^–1^ DW)
(9)  Chloroplast metabolite level:
  $$\frac{X_{\text{P}}}{EP_{\text{P}}}$$
  [µmol/(µmol mg^–1^ DW)]
(10)  Extra-chloroplast metabolite level:
  $$\frac{X_{\text{C}}}{EC_{\text{C}}}$$
  [µmol/(µmol mg^–1^ DW)]

The contents of the total leaf samples and the contents of the non-aqueous chlorophyll fraction can therefore be written as follows:

$$M_{\text{T }}=\;\frac{X_{\text{P}}}{EP_{\text{P}}}EP_{\text{T}}+\frac{X_{\text{C}}}{EC_{\text{C}}}EC_{\text{T}}$$ (1)

$$M_{\text{Cf}}\;=\;\frac{X_{\text{P}}}{EP_{\text{P}}}EP_{\text{Cf}}+\frac{X_{\text{C}}}{EC_{\text{C}}}EC_{\text{Cf}}$$

By resolving equations (1) and (2), it is possible to determine the unknowns,

$$\frac{X_{\text{P}}}{EP_{\text{P}}}$$

and

$$\frac{X_{\text{C}}}{EC_{\text{C}}}$$,

and then the actual metabolite levels in the extra-chloroplast compartments and in the chloroplasts can be obtained (expressed in terms of per unit of chlorophyll). The metabolite level in the extra-chloroplast compartment, *M*_EX_, was obtained as follows:

$$M_{\text{EX}}\left(\text{r}.\text{u}.µ{\text{g}}^{–\text{1}}\text{DW}\right)=\frac{\frac{X_{\text{C}}}{EC_{\text{C}}}*EC_{\text{T}}}{Y_{\text{T}}}$$

and the metabolite level in the chloroplast, *M*_C_, was calculated as:

$$M_{\text{C}}\left(\text{r}.\text{u}.µ{\text{g}}^{–\text{1}}\text{DW}\right)=\frac{\frac{X_{\text{P}}}{EP_{\text{P}}}*EP_{\text{Cf}}}{Y_{\text{Cf}}}$$

### Statistics

Data were compared pairwise using Students *t*-test. Significant differences in data groups were calculated with Fisher’s LSD (with *P*<0.05) (ANOVA, InfoStat).

## Results

### Gas exchange and photosynthetic quantum yield

**Fig 1.**
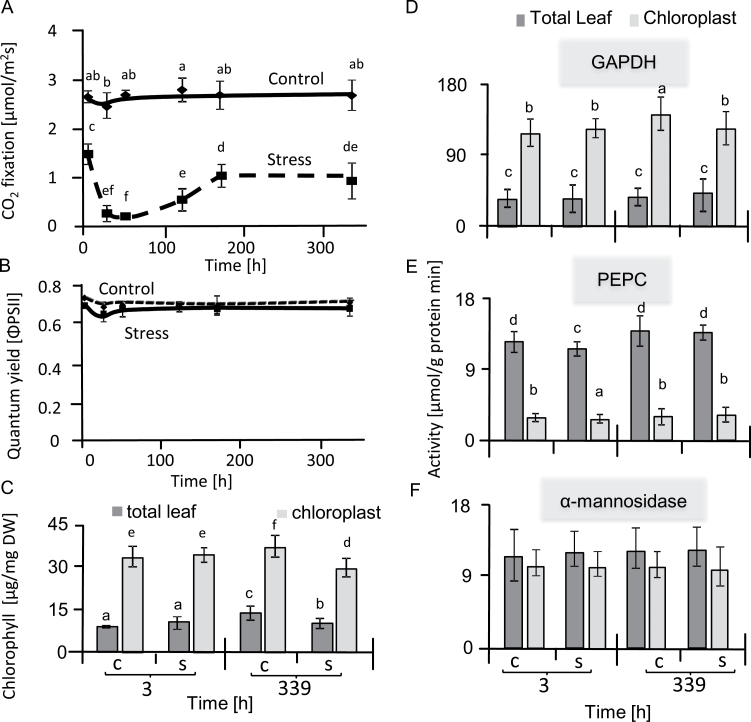
State of photosynthesis and distribution of markers between the non-aqueous chloroplast fraction and the total sugar beet leaf. (A) CO_2_ fixation rates of the second and third leaf pairs were measured by infrared gas analysis, beginning when the salt stress was increased to its maximum level of 300 mM NaCl. Data are means ±SD of *n*=5 experiments. Different letters indicate significant differences as determined using Fisher’s LSD (*P*<0.05). (B) Quantum yield of photosystem II as measured with a pulse amplitude-modulated chlorophyll fluorescence analysis system in parallel with CO_2_ fixation. (C) Total chlorophyll contents. (D–F) Activities of NADP-glyceraldehyde-3-phosphate dehydrogenase (GAPDH) (D), phospho*enol*pyruvate carboxylase (PEPC) (E), and α-mannosidase (F), in the non-aqueous chloroplast fraction and the total leaf sample of salt-stressed and control sugar beet. Data are means ±SD of *n*=6 experiments. Different letters indicate significant differences as determined using Fisher’s LSD (*P*<0.05). C, control; S, salinity.

CO_2_ assimilation rate and photosynthetic quantum yield (ΦPSII) were determined in sugar beet leaves under salt stress and control conditions (Fig. 1). CO_2_ assimilation significantly decreased in stressed leaves with the lowest rate being obtained at 51 h of salt treatment. Assimilation then started to recover and reached about 40% of the control level after one week of treatment and stayed constant until the end of the experiment (Fig. 1A). CO_2_ assimilation rates of control plants remained high throughout the experiment. Significant differences in ΦPSII were not observed between stressed and control plants, providing evidence of efficient regulation of primary reactions of photosynthesis during salt stress (Fig. 1B). Sugar beet leaves were thus apparently able to efficiently balance light-harvesting, redox adjustment of electron transport, energy dissipation by non-photochemical mechanisms, and energy consumption in metabolism so that photosystem II maintained a functional state under high salinity that was similar to the control conditions. Therefore, in the next step we examined the metabolic state of the chloroplasts in the leaves by subcellular fractionation.

### Enrichment of chloroplasts by non-aqueous extraction

Non-aqueous fractionation of freeze-dried sugar beet leaves was performed successfully, and yielded a 4-fold enrichment of chlorophyll contents compared to the whole leaf (Fig. 1C). According to Heber (1957), chlorophyll contents above 30 µg mg^–1^ dry weight indicate close to maximal enrichment of the chloroplasts. The chloroplast fraction also showed a significant increase in NADP-glyceraldehyde 3-P dehydrogenase activity compared to whole-leaf fractions (Fig. 1D). Both NADP-GAPDH and chlorophyll are exclusively found in the chloroplast. Thus, the similarly high enrichment of both markers confirmed the successful non-aqueous purification of the chloroplasts. The cytosolic enzyme phosphoenolpyruvate carboxylase (PEPC) catalyses the carboxylation of phosphoenolpyruvate to oxaloacetate using carbonate as a co-substrate to produce oxaloacetate. PEPC was determined by measuring NADH oxidation using malate dehydrogenase.

$$\text{PEP}+{\text{HCO}}_{\text{3}}{}^{–}\to \text{Oxaloacetate}+\text{NADH}+P_{i}\to \text{Malate}+{\text{NAD}}^{+}+P_{i}$$

The activity of PEPC showed that the chloroplast fractions were largely free from contamination by the cytosol (Fig. 1E). The combined results from the quantification of the cytosolic and chloroplast markers revealed that the chloroplast fraction was highly enriched in chloroplast-specific components by more than 14-fold, which provided an excellent basis for further detailed analysis. α-Mannosidase activity is commonly used as vacuolar marker, although it is also associated with the endomembrane system and cell walls (Van Der Wilden and Chrispeels, 1983; Liebminger *et al.*, 2009). The activity of α-mannosidase was lower in the chloroplast fraction than in the whole leaf (Fig. 1F).

### Metabolite changes in leaves of salt-stressed sugar beet

**Fig 2.**
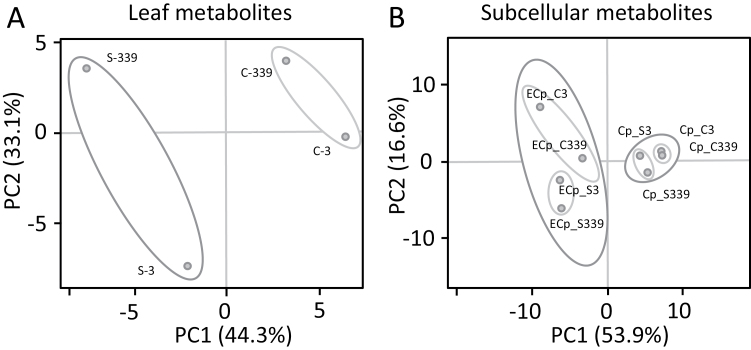
Principal component analysis (PCA) of metabolites detected in whole leaves (A) and subcellular compartments of sugar beet (B) under control (labeled ‘C’) and salt-stressed (labeled ‘S’) conditions. The numbers ‘3’ and ‘339’ indicate the harvest time points (h) beginning when the salt stress was increased to its maximum level of 300 mM NaCl. ‘Cp’ stands for chloroplast, and ‘ECp’ for extra-chloroplast space. Unit variance scaling was applied to rows; singular value decomposition (SVD) with imputation was used to calculate the principal components. Data for 83 metabolites in the form of means ±SD of *n*=6 experiments were used for this PCA. The data were distributed normally.

Metabolites were profiled in sugar beet leaves of control and salt-stressed plants exposed to 300 mM NaCl for 3 h and 14 d. Principal component analyses (PCA) carried out for both time points revealed the dynamics of the metabolic adjustment (Fig. 2). *A priori* testing determined that the data were normally distributed. The metabolic state of salt-stressed leaves at 3 h was strongly separated from that of control plants (Fig. 2). At 14 d, the contributions of the principal components shifted so that there was less contribution of PC1 and more of PC2. This trend was apparent in both control and salt-stressed leaves, but was much more pronounced in the latter. Separation according to PCA was also seen in the subcellular fractions. The chloroplast metabolites clustered in close proximity, while the extra-chloroplast metabolite patterns showed a profound and clear separation (Fig. 2). The results overall hint at two distinct responses, namely an ageing effect and a stress effect (see Supplementary Table S3).

**Fig. 3.**
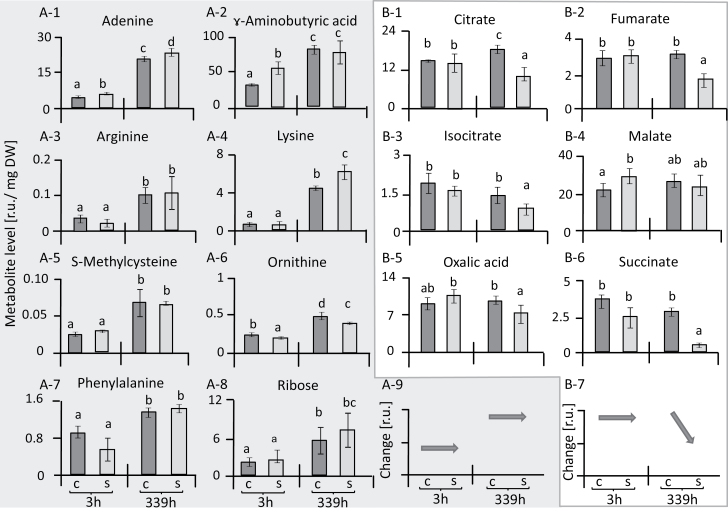
Changes in metabolites in leaves of sugar beet under salt-stress and control conditions. Metabolites are expressed in terms of relative units (r.u.). Parts A-1 to A-8 show a pattern of response that is strongly linked to ageing, and this is summarized in A-9. Organic acids in parts B-1 to B-6 showed little response during early salt stress but were down-regulated at 339 h; this pattern is summarized in B-7. Data are means ±SD of *n*=6 experiments. Different letters indicate significant differences as determined using Fisher’s LSD (*P*<0.05). C, control; S, salinity.

**Fig. 4.**
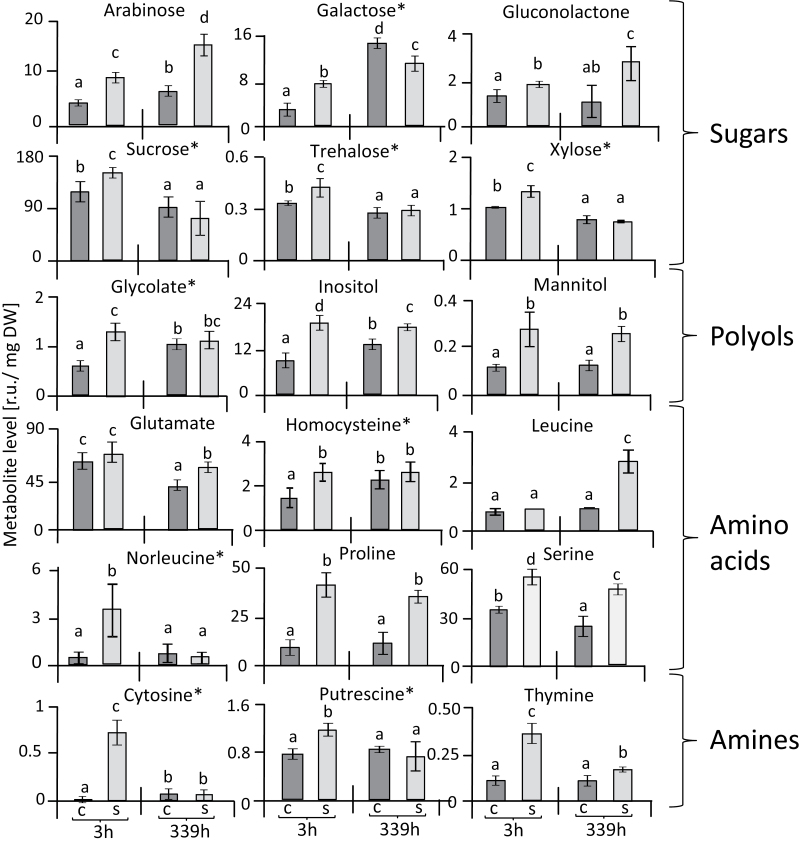
Metabolites in leaves of sugar beet showing up-regulation in response to salinity stress. Metabolites are expressed in terms of relative units (r.u.), and are divided into four groups, as indicated. With the exception of leucine and glutamate, all the metabolites showed an increase at 3 h in response to salt stress. Nine metabolites (indicated by *) were increased after 3 h but not at 339 h in response to salt stress. Data are means ±SD of *n*=6 experiments. Different letters indicate significant differences as determined using Fisher’s LSD (*P*<0.05). C, control; S, salinity.

**Fig. 5.**
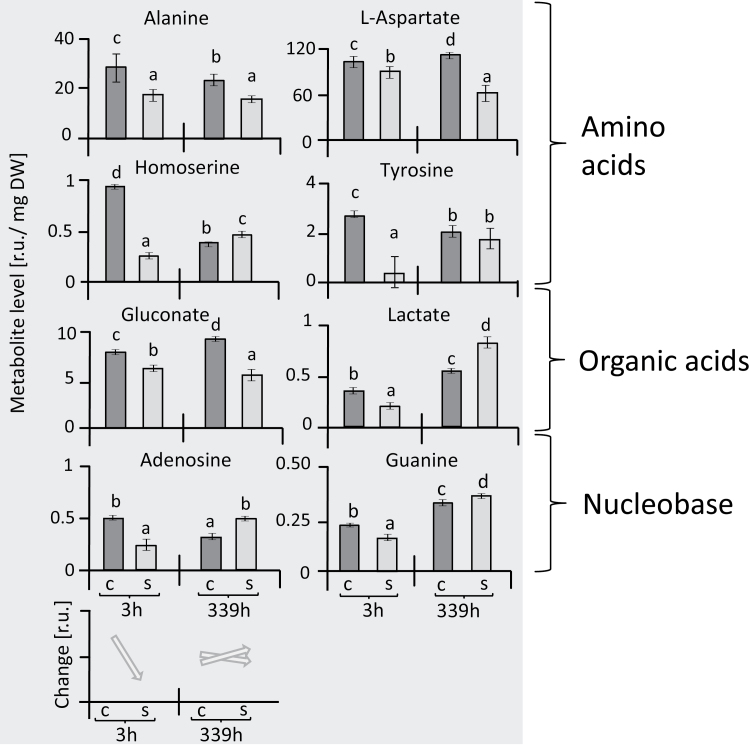
Changes in metabolites in leaves of sugar beet showing initial down-regulation under salinity stress. Metabolites are expressed in terms of relative units (r.u.), and the patterns of change are summarized at the bottom of the figure. Data are means ±SD of *n*=6 experiments. Different letters indicate significant differences as determined using Fisher’s LSD (*P*<0.05). C, control; S, salinity.

Categorizing the responses of individual metabolites revealed a set that showed a strong age-dependent increase over the period of 14 d in both the stressed and the control plants, namely adenine, arginine, ɤ-aminobutyric acid (GABA), lysine, ornithine, phenylalanine, and ribose (Fig. 3A1–8). This group was of relatively little interest for understanding salt acclimation since the age-related response also occurred in control plants. The metabolites that were stress-related showed distinct types of dynamics. One set comprised organic acids that were little affected after 3 h of reaching maximal salt stress and then, except for malate, decreased after a further 2 weeks of salinity (Fig. 3B1–7). Another set of metabolites, including sugars, polyols, amino acids, and amines, showed a different response to salinity stress (Fig. 4). Seven of these metabolites showed significant accumulation at both 3 h and 14 d after maximal salt stress was applied, namely arabinose, gluconolactone, inositol, mannitol, proline, serine, and thymine. In contrast, nine metabolites, namely galactose, sucrose, trehalose, xylose, norleucine, putrescine, cytosine, homocysteine, and glycolate, were initially increased but dropped or were unaltered at the later time point (Fig. 4). Finally, lactate, homoserine, adenosine, and guanine initially showed lower levels, but their responses at the later time point of salinity stress were variable (Fig. 5).

### Subcellular compartmentation of metabolic changes

**Fig. 6.**
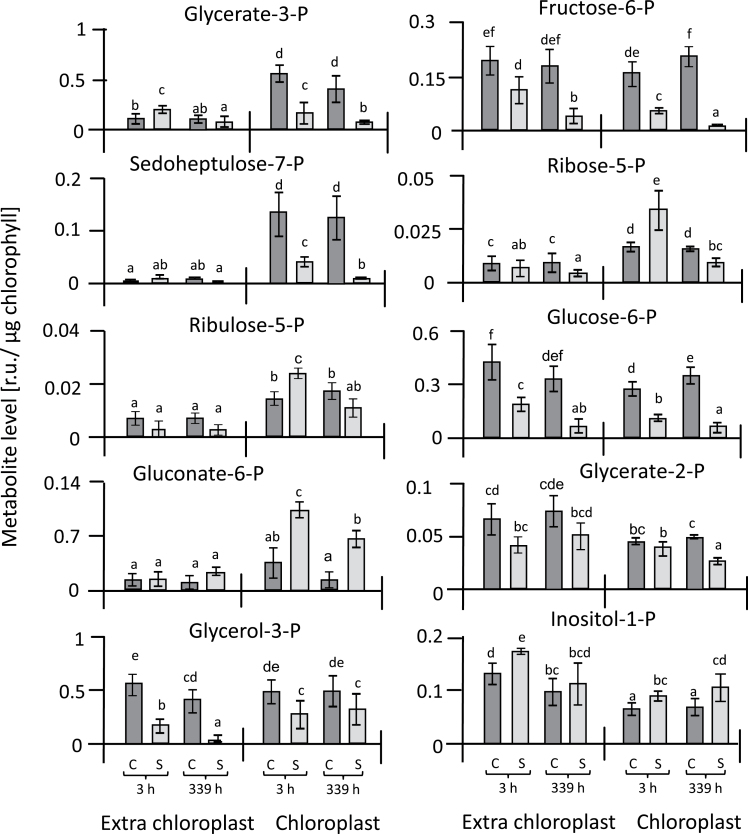
Changes in contents of phosphorylated metabolites in chloroplasts and the extra-chloroplast space of sugar beet under salinity stress and control conditions. Metabolites are expressed in terms of relative units (r.u.). Data are means ±SD of *n*=6 experiments. Different letters indicate significant differences as determined using Fisher’s LSD (*P*<0.05). C, control; S, salinity.

**Fig. 7.**
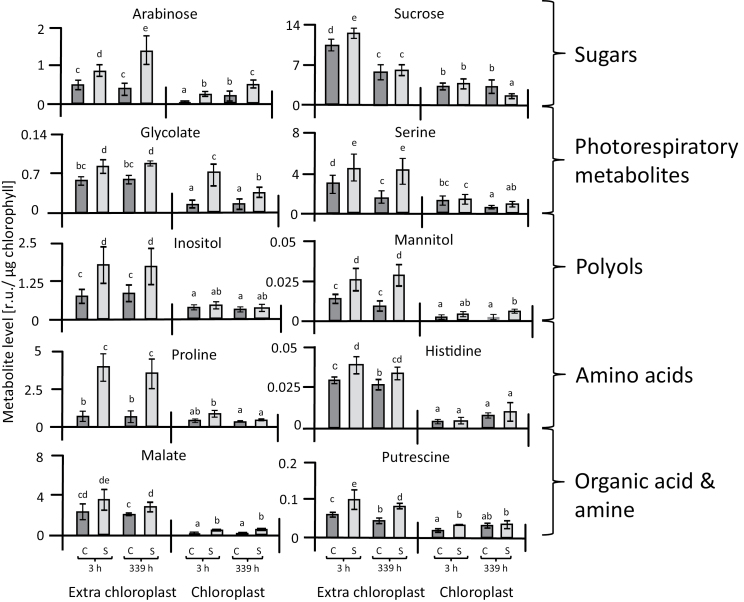
Changes in contents of different metabolites in chloroplasts and the extra-chloroplast space of sugar beet under control and salinity stress. Metabolites are expressed in terms of relative units (r.u.). Data are means ±SD of *n*=6 experiments. Different letters indicate significant differences as determined using Fisher’s LSD (*P*<0.05). C, control; S, salinity.

**Fig. 8.**
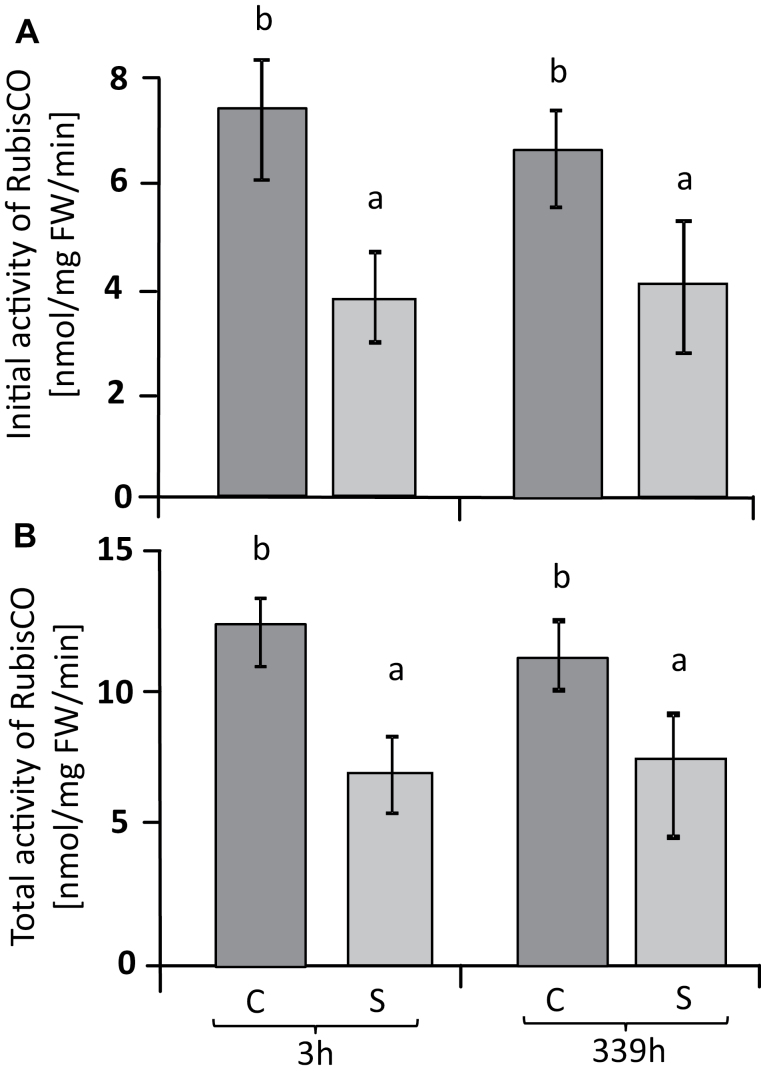
Rubisco activity of sugar beet leaves under salinity and control conditions. Initial activity (A) and total activity (B). Data are means ± SD of *n*=6. Different letters indicate significant differences as determined using Fisher’s LSD (*P*<0.05). C, control; S, salinity.

The isolation of chloroplasts by non-aqueous fractionation allowed the calculation of metabolite concentrations in subcellular compartments. The advantage of the non-aqueous fractionation technique compared to the frequently used method of continuous gradients (Gerhardt and Heldt, 1984; Wienkoop *et al.*, 2010) is the high enrichment of chloroplasts by three types of centrifugation: firstly centrifugation at an equilibrium density, and then two sedimentation steps that remove both the heavier and thus faster-sedimenting cell constituents, and also the lighter and thus slower-sedimenting ones. The fraction obtained was more than 14-fold enriched in chloroplasts and thus represented a very homogeneous extract. Further correction for cross-contamination provided access to precise information on chloroplast metabolite concentrations. The results of the correction were first examined by assessing the distribution of phosphorylated metabolites that are present in two compartments, the cytosol and the chloroplast. Among the detected metabolites, the Calvin–Benson cycle (CBC) constituent sedoheptulose-7-P was almost exclusively associated with the chloroplast, thus confirming the precision of the isolation of the chloroplasts by the non-aqueous fractionation (Fig. 6). Other CBC metabolites identified included glycerate-3-P, fructose-6-P, ribose-5-P, and ribulose-5-P, which are also part of the glycolysis and oxidative pentose phosphate pathways. Ribose-5-P, ribulose-5-P, and gluconate-6-phosphate were the metabolites that accumulated in the chloroplast under salinity stress (Fig. 6, Supplementary Table S3). Arabinose, glycolate, inositol, malate, mannitol, and putrescine accumulated in the chloroplasts of salt-stressed leaves relative to those of control plants (Fig. 7). Essentially the same set of metabolites (arabinose, glycolate, inositol, malate, mannitol, proline, putrescine, and serine) were also highly abundant in the extra-chloroplast fraction under salinity stress (Fig. 7, Supplementary Table S3). To link these biochemical alterations, we analysed the initial and total activity of Rubisco (Fig. 8). Initial Rubisco activity in salt-treated plants was lower than in controls, with slightly higher inhibition after 3 h (49%) than after 14 d (39%). Inhibition of initial activity was slightly greater than inhibition of total activity both at 3 h and 14 d (40% and 32%, respectively), indicating several levels of regulation of Rubisco activity under salinity stress.

## Discussion

### State of photosynthesis and carbon assimilation

Photosynthetic CO_2_ fixation dropped upon exposure to salt stress. This inhibition is a common phenomenon of severe salt stress and has several causes. Diffusive limitations caused by stomatal closure and metabolic limitations resulting from down-regulation of photosynthetic electron transport and the carbon cycle, and also by ionic inhibition (Flexas *et al.*, 2004), decrease photosynthetic efficiency and thus biomass production. Calvin cycle activity is a major target of salinity toxicity and overexpression of seduheptulose-1,7-bisphosphatase stimulates growth in rice under stress (Feng *et al.*, 2007). The results of our metabolite analysis showed a differential effect of salinity on the state of the Calvin cycle in sugar beet. Metabolites in the cycle following carboxylation, such as 3-phosphoglycerate, fructose-6-phosphate, and also sedoheptulose-7-phosphate, were depleted in salt-stressed chloroplasts. In contrast, the metabolites used in the regeneration pathway of ribulose-1,5-bisphosphate, namely ribose-5-phosphate and ribulose-5-phosphate, were highly accumulated. This indicates inhibition of the primary carboxylation reaction in salt-stressed sugar beet. In line with this assumption, initial Rubisco activity was more inhibited than its total activity under salt-stress conditions compared with control plants. Thus, apart from diffusion limitation by closed stomata, Calvin cycle activity was down-regulated biochemically. It is noteworthy that photosynthetic carbon assimilation continued despite the accumulation of more than 500 mmol l^–1^ Na^+^ and 350 mmol l^–1^ Cl^–^ in the leaf tissue after 14 d in 300 mmol l^–1^ NaCl (Hossain *et al.*, 2017). However, as shown by the subcellular analysis, the Calvin cycle adopted a different regulatory state as indicated by the shifts in relative metabolite levels. It should be noted that for a complete and quantitative analysis of Calvin–Benson cycle intermediates, in particular of organic diphosphates, other analytical approaches such as liquid chromatography/mass spectrometry and isotope labeling should be considered in future work (Arrivault *et al.*, 2015); however, the data presented here reliably describe the relative changes of monophosphates among the samples.

Phosphorylated metabolites that are exclusively distributed between the cytosol and chloroplast were reliably determined in the chloroplast fraction (Fig. 6). It was shown previously that the determination of the exact distribution of non-phosphorylated metabolites is less reliable in a two-fraction system because they are distributed among several compartments (Heber, 1957). But the 6-fold enrichment of chloroplasts over the vacuolar space (Table 1) indicates that the calculated pool sizes reflect the alterations in the compartments *in vivo* and allow reliable metabolite distributions between chloroplast and extra-chloroplast compartments to be obtained. The increase in glycolate and serine under salinity was pronounced, particularly after 3 h, and provides evidence of the stimulation of photorespiration under salt stress. Up-regulation of photorespiration plays an important role in abiotic stress responses, both to dissipate excess reducing energy in order to prevent over-reduction of the photosynthetic electron transport chain and to provide metabolites to other compartments and pathways (Voss *et al.*, 2013). Glycolate metabolism in photorespiration recovers 75% of the carbon from phosphoglycolate, efficiently removes potent inhibitors of photosynthesis, and protects the chloroplast under stress conditions (Kebeish *et al.*, 2007; Hossain and Dietz, 2016). Photorespiration is considered to be more efficient than the Mehler reaction in preventing the over-reduction of the electron transport chain (Wu *et al.*, 1991; Heber *et al.*, 1996).

**Table 1.** Summary of chloroplast enrichment in non-aqueous chloroplast fractions. The marker activity in the chloroplast fraction was divided by the marker activity in the whole leaf. The chlorophyll enrichment factor (EF) was lower than that of GAPDH-EF due to some chlorophyll losses during fractionation in organic solvents. The chloroplast fractions were depleted relative to the cytoplasm. Data are means, *n*=6

	Control 3 h	Stress 3 h	Control 14 d	Stress 14 d
Chloroplast EF (Chlorophyll)	3.7	3.3	2.7	2.9
Chloroplast EF (GAPDH)	5.1	5.1	5.7	4.8
Cytoplasm EF (PEPC)	0.32	0.29	0.31	0.34
Chloroplast EF /Cytoplasm EF	16.0	17.3	18.2	14.3
Vacuole EF (α-mannosidase)	0.89	0.86	0.86	0.80
Chloroplast EF/ Vacuole EF	5.7	5.9	6.7	6.0

Levels of glucose-6-phosphate, fructose-6-phosphate, glycerate-2-phosphate, and glycerol-3-phosphate outside the chloroplast were greatly decreased in salt-stressed plants, which indicated down-regulation of glycolysis as observed before in barley grown under salt-stress, but without subcellular resolution (Wu *et al.*, 2013). Taken together, the observed patterns of changes in phosphorylated metabolites is indicative of down-regulation of central energy metabolism.

### Osmotic adjustment and compatible solutes in sugar beet

Changes in specific metabolite pools, especially sugars, amino acids, and organic acids, can be considered as hallmarks of the salinity stress response in plants (Sanchez *et al.*, 2008). The active accumulation of osmolytes increases the osmotic potential of plasmatic compartments such as the cytosol, matrix, and stroma, and maintains proper water relations and cell turgor. They also participate in scavenging ROS, protect protein structures and membranes, and consume excess reducing power (Parida *et al.*, 2004; Ahmad *et al.*, 2013). Interestingly, not all plants accumulate the same components of compatible solutes, and osmolytes such as glycine betaine and trehalose are only generated by a limited number of species (Krasensky and Jonak, 2012).

The increased abundance of sugars and sugar derivatives such as arabinose, inositol, mannose, sucrose, trehalose, xylose, and galactose under salinity stress is in line with the need to accumulate compatible solutes in plasmatic compartments as vital salt-tolerance mechanisms (Gao *et al.*, 1998; Aquino *et al.*, 2011). Reducing sugars such as sucrose and non-reducing sugars such as trehalose (α-D-glucopyranosyl-1,1-α-D-glucopyranoside) function as stress protectants, stabilizing proteins and membranes and protecting them from denaturation (Ge *et al.*, 2008; Paul *et al.*, 2008). Starch, as the main carbohydrate store in most plants, can be rapidly mobilized to provide soluble sugars. Its metabolism is very sensitive to changes in environmental stress, often allowing the accumulation of soluble sugars in leaves at the expense of depletion of the starch pool (Todaka *et al.*, 2000; Basu *et al.*, 2007; Kempa *et al.*, 2008).

Polyols with multiple hydroxyl groups function as compatible solutes, but also as low molecular-weight chaperones and ROS-scavenging compounds (Parida and Das, 2005; Saxena *et al.*, 2013). Levels of mannitol and, in particular, of inositol increased in salt-stressed sugar beet in the extra-chloroplast compartments (Fig. 7). Mannitol is a major photosynthetic product in many higher plants and enhances the tolerance to salt stress primarily through osmotic adjustment (Iwamoto and Shiraiwa, 2005). Mannitol also enhances tolerance to salinity and water deficit by scavenging hydroxyl radicals and stabilizing macromolecules (Abebe *et al.*, 2003; Chen *et al.*, 2005). Moreover, in tobacco, mannitol protects thioredoxin, ferredoxin, glutathione, and the thiol-regulated enzyme phosphoribulokinase from the effects of hydroxyl radicals (Shen *et al.*, 1997). In the present study, mannitol contents doubled under salinity in sugar beet but total accumulation was low, even when considering the preferential extra-chloroplast accumulation. The cyclic polyol inositol and its methylated derivatives play a protective role in plants and increase tolerance to salt stress (Patra *et al.*, 2010; Sengupta *et al.*, 2008; Majee *et al.*, 2004; Sheveleva *et al.*, 1997). Inositol amounts increased in the extrachloroplast compartments under salinity in sugar beet. Thus, both mannitol and inositol may contribute to the high fitness of sugar beet under salinity.

Accumulation of nitrogen-containing compounds may indicate either a role in conferring salinity tolerance to sugar beet or a deregulation of nitrogen metabolism, or both. Increased levels of proline and putrescine often accompany the response of plants to abiotic stresses (Zuther *et al.*, 2007; Kempa *et al.*, 2008; Sanchez *et al.*, 2008; Lugan *et al.*, 2010), and have a beneficial effect on cell viability under salinity (El-Shintinawy and El-Shourbagy, 2001; Widodo *et al.*, 2009). Levels of proline increased several fold in response to salinity stress in sugar beet. Proline not only provides tolerance toward stress but also serves as an organic nitrogen reserve during stress recovery. Its accumulation usually arises in the cytosol where it contributes substantially to the cytoplasmic osmotic adjustment (Hoque *et al.*, 2008). The correction for cross-contamination accentuated the cytosolic localization of the salinity-induced proline pool in sugar beet. Proline functions as an osmolyte, a ROS scavenger, and a molecular chaperone that stabilizes the structure of proteins; it thus protects cells from damage during stress, enhances antioxidant defense mechanisms, and minimizes the development of damage (Funck *et al.*, 2008; Verbruggen and Hermans, 2008; Szabados and Savouré, 2010). Amines are ubiquitously occurring low molecular-weight cationic molecules that are widely distributed throughout the plant kingdom and play various roles in tolerance to abiotic stress including salinity (Groppa and Benavides, 2008). The diamine putrescine is the smallest polyamine and is synthesized from ornithine or arginine by ornithine decarboxylase or arginine decarboxylase, respectively (Slocum *et al.*, 1984; Alcázar *et al.*, 2010). Our results showed an increase in putrescine levels in salt-stressed sugar beet, mostly in the extra-chloroplast space. Accumulation of putrescine in response to salinity stress in sugar beet was also reported by Quinet *et al.* (2010). Putrescine is one of the most important polyamines as it, stabilizes DNA under salinity stress, including in the mitochondria and chloroplasts. In addition, it stimulates many steps of protein biosynthesis through interactions with nucleic acids and stabilizes biomembranes (Sairam and Tyagi, 2004). An increase in polyamine levels seemed to stabilize the photosynthetic apparatus under salt stress (Shu *et al.*, 2015).

Malic acid plays an important role in the exchange of reducing power between different subcellular compartments, e.g. between chloroplasts and the cytosol, if the reducing power from photosynthetic electron transport exceeds consumption in the dark reaction (Scheibe *et al.*, 2005). Stimulation of chloroplast malate dehydrogenase and an increased capacity of the malate valve allow for correct maintenance of the redox homeostasis within and between the different cell compartments (reviewed by Hossain and Dietz, 2016). The increased malate levels observed under salinity may indicate increased exchange of reducing power via the malate valve in response to salinity. Moreover, the increased concentrations of malate, proline, and sucrose in salt-stressed sugar beet, mostly in the extra-chloroplast space, provide an indication that they serve as additional major osmolytes during adaptation to salt stress, in addition to polyols.

**Fig. 9.**
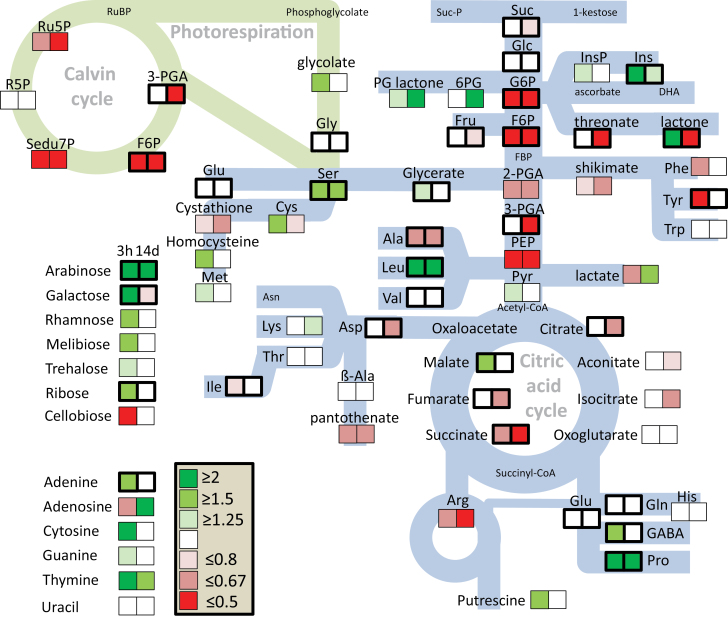
Summary of metabolic changes in sugar beet leaves under salt stress relative to control conditions. For each pair of boxes, the one on the left gives the change at 3 h of stress (beginning when the salt stress was increased to its maximum level of 300 mM NaCl) and the one on the right hand is for 14 d. Boxes bordered by thick lines indicates metabolite levels >2 ru mg^–1^ fresh weight, those bordered by thin lines indicate ≤2 ru mg^–1^ (ru, relative units). Green indicates up-regulation and red indicates down-regulation under salt stress. Abbreviations: 6PG, 6-phospho glucono lactone; F6P, fructose-6-phosphate; Fru, fructose; Glc, glucose; Ins, inositol; PEP, phosphoenol pyruvate; PGA, phosphoglycerate; PGlactone, phosphoglucono lactone; Pyr, pyruvate; R5P, ribulose-5-phosphate; Ru5P, ribulose-5-phosphate; Sedu7P, seduheptulose-7-phosphate. Amino acids are abbreviated to standard three-letter codes.

**Fig. 10.**
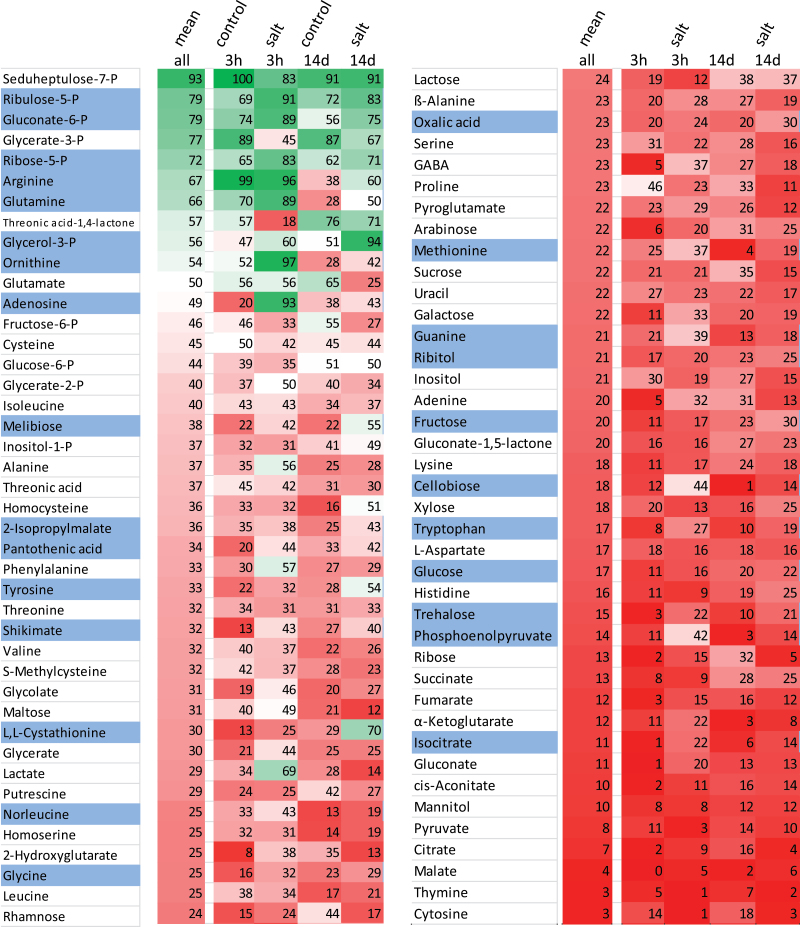
Subcellular localization of metabolites. The metabolite distributions from all treatments were averaged, % chloroplast localization was calculated and the values sorted from highest to lowest (first column, ‘mean’). The same calculation was then made for each treatment, i.e. 3 h control and salt-stressed, and 14 d control and salt-stressed. The darkest shade of green indicates 100% chloroplast localization (only seen for seduheptulose-7-phosphate in 3 h control) and the darkest shade of red indicates 100% extra-chloroplast localization. Blue shading for a metabolite indicates a shift to more chloroplast localization under salt stress at both time points.

Our previous investigation of the antioxidant system using the same experimental set-up revealed lower accumulation of ROS in sugar beet exposed to 300 mM NaCl compared with non-stressed controls (Hossain *et al.*, 2017). Efficient up-regulation of antioxidant enzymes and alternative oxidases, and down-regulation of NADPH oxidases provide a framework for understanding this peculiar ability to suppress ROS accumulation (Hossain *et al.*, 2017). The maintenance of redox- and ROS-homeostasis appears to be an important element of the salt-stress tolerance syndrome. Our current study provides additional clues on the metabolic changes at the subcellular level that accompany acclimation to salinity stress (Fig. 9). At the whole-leaf level, metabolites of the Calvin–Benson cycle, glycolysis, the citric acid cycle, and shikimate-derived amino acids decreased, while certain peripheral metabolites such as proline, GABA, inositol, and leucine accumulated. It appears that the Calvin cycle shifts to a different regulatory state with a high control by Rubisco. Concerning the subcellular distribution (Fig. 10), several metabolites shifted to a preferred localization in the chloroplasts, in particular ribose-5-phosphate, ribulose-5-phosphate, and gluconate-6-phosphate among the detected phosphorylated metabolites, and shikimate, melibiose, ornithine, glutamine, and trehalose. Generally, high amounts of glycolate and serine have reflected enhanced photorespiration under salinity. Levels of compatible solutes such as proline, arabinose, mannitol, inositol, and putrescine, which have beneficial effects on biochemical processes, increased in the extra-chloroplast space. Sugars and amino acids accumulated under salt stress, indicating an increase in carbon and nitrogen availability probably at the expense of growth.

## Supplementary data

Supplementary data are available at *JXB* online.

Fig. S1. Outline of the experimental design.

Fig. S2. Detailed procedure of non-aqueous fractionation of chloroplasts.

Table S1. List of all detectable metabolites in sugar beet as determined by GC-MS.

Table S2. Changes in metabolite in whole leaves of sugar beet in response to salt stress.

Table S3. Changes in metabolites in different fractions of sugar beet leaves in response to salt stress.

## Author contributions

MSH designed the study, conducted the experiments, measurements, calculations, analyses, and wrote the paper. MP and JK performed and evaluated the GC-MS measurements. AIE helped during the experimental set-up. KJD designed and guided the study, developed the equations for the calculations, discussed the data, and wrote the paper.

## Supplementary Material

supplementary_figures_S1_S2_Tables_S1_S3
