# Supplementary material for: Metabolite profiling at the cellular and subcellular level reveals metabolites associated with salinity tolerance in sugar beet
Source: J Exp Bot. 2017 Nov 11;68(21-22):5961–76. doi: 10.1093/jxb/erx388 (PMC5854137; doi:10.1093/jxb/erx388)
Supplement: supplementary_figures_S1_S2_Tables_S1_S3 [file erx388_suppl_supplementary_figures_s1_s2_tables_s1_s3.pdf]

Supplementary Table S1: List of all detectable metabolites in sugarbeet by gas chromatography-mass spectrometry (GC-MS).

**Compounds**	**Quantification mass [m/z]**	**Compounds**	**Quantification mass [m/z]**
Glucose	319	L,L-Cystathionine	218,278
Fructose	307	Homocysteine	128,234
Galactose	319	Phenylalanine	192
Lactose	361	Arginine	157,256
Sucrose	361	Histidine	154
Cellobiose	361	Lysine	156
Maltose	480	Tyrosine	218
Trehalose	361	Tryptophan	202
Melibiose	204,361	Glutamine	156
Arabinose	217,307	Glutamate	230,246
Ribose	217	Ornithine	142
Rhamnose	277	ɤ-Aminobutyric cid(GABA)	174,304
Glucose-6-P	387	Pyruvate	174
Fructose-6-P	315	Lactate	191
Ribose-5-P	315,299	Glycolate	177
Ribulose-5-P	357	Succinate	247,409
Sedoheptulose-7-P	387,471	Fumarate	245
Glycerol-3-P	357	Malate	245,307
Glycerate-2-P	299,315,459	Pyrogultamate	230
Glycerate-3-P	227,299,459	L-Aspartate	100,188,232
Glycerate	189,192	Citrate	257
Gluconate	333	Isocitrate	245,319
Gluconat-6-P	333,387	cis-Aconitate	229
Xylose	217,307	2-Isopropylmalate	275
Ribitol	217	a-Ketoglutarate	198
Mannitol	217,319	2-Hydroxyglutarate	203,247
Inositol	305	Phosphoenolpyruvate	369
Inositol-1-P	318	Oxalic acid	133
Alanine	116	Gluconate-1,5-lactone	129,220
Valine	144	Pantothenic acid	201
Leucine	158	Threonic acid	292
Isoleucine	158	Threonic acid-1,4-lactone	217,247
Proline	142	Adenine	264
Glycine	174	Adenosine	236
Norleucine	158	Guanine	352
Serine	204	Thymine	255
Threonine	101	Uracil	255,241
S-Methylcysteine	218	Cytosine	240,257
ß-Alanine	248	Putrescine	174
Homoserine	218	Shikimate	204
Methionine	176	Dopamine	174
Cysteine	220		

Supplementary Fig. S1: Outline of the experimental design.
(A) Stepwise daily up-salting to 300 mM NaCl and harvest after 3h, or 339h (14d period). (B) Phenotype of plants growing in hydroponics.

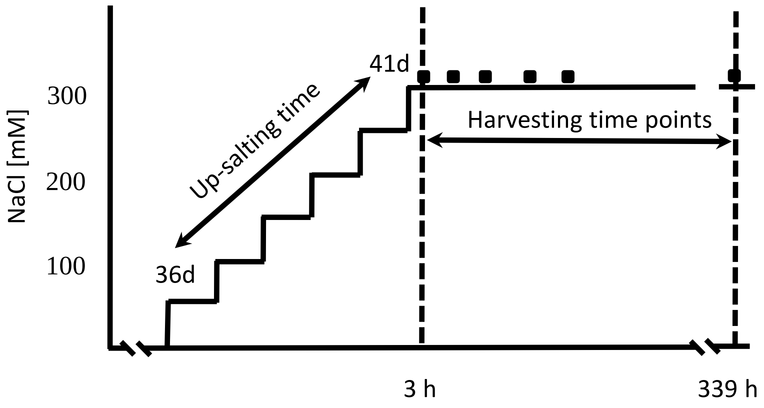

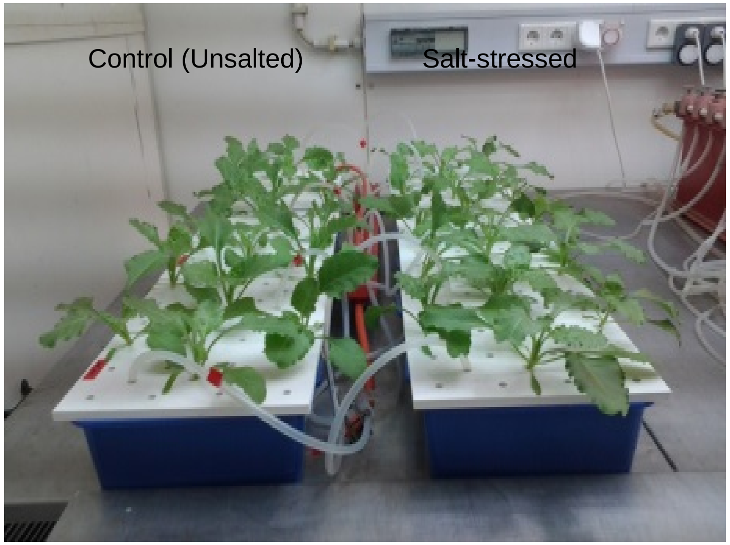

Supplementary Fig. S2: Schematic representation of the experimental procedure for non-aqueous isolation of chloroplast.

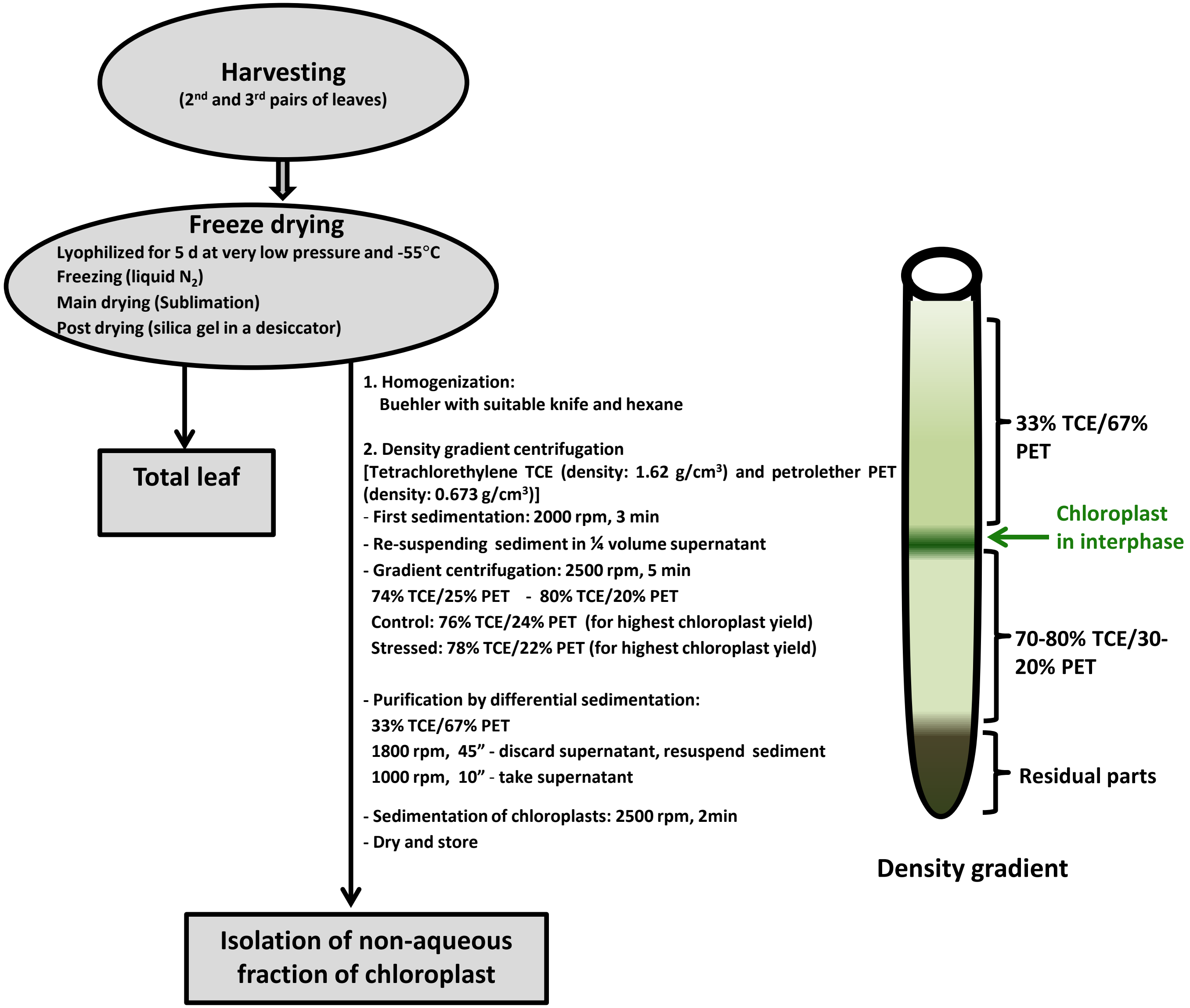

Supplementary Table S2: Metabolites changes in total leaf of sugarbeet. Data are means ±SD of n=6 experiments. Data groups of significant difference were assessed with Fisher's LSD ($p<0.05$) and labelled with different letters.

	Metabolites	[r.u./ mg DW]			
		Control 3h	Stress 3h	Control 339h	Stress 339h
Sugars	Glucose	58.0±2.15ab	61.4±2.42c	60.3±2.71bc	55.1±2.21a
	Fructose	33.4±1.41b	34.0±0.14b	36.1±1.85c	27.7±1.03a
	Galactose	3.11±1.24a	7.49±0.56b	14.2±0.54d	11.3±0.23c
	Lactose	0.136±0.004a	0.323±0.034d	0.201±0.004c	0.165±0.009b
	Sucrose	130.7±12.12b	156.7±2.94c	109.5±14.84a	78.86±15.83a
	Cellobiose	0.2327±0.0020c	0.1119±0.0560a	0.1854±0.0002b	0.1997±0.0097bc
	Maltose	0.064±0.007b	0.051±0.004a	0.073±0.006c	0.068±0.006bc
	Trehalose	0.30±0.02b	0.39±0.03c	0.26±0.01a	0.26±0.01a
	Melibiose	0.52±0.10a	0.85±0.09b	0.73±0.10b	0.77±0.09b
	Arabinose	4.28±0.02a	9.68±1.03c	6.56±0.94b	16.6±2.26d
	Ribose	2.31±0.76a	3.18±1.08a	6.18±2.23b	7.60±2.80bc
	Rhamnose	0.107±0.001a	0.156±0.002b	0.160±0.019b	0.189±0.007c
	Xylose	1.064±0.003b	1.467±0.108c	0.813±0.051a	0.821±0.012a
	Ribitol	16.22±0.406a	18.83±0.013d	16.83±0.003b	18.27±0.004c
	Mannitol	0.121±0.007a	0.284±0.080b	0.129±0.427a	0.269±0.217b
	Inositol	9.22±1.33a	19.9±1.53d	13.7±0.02b	18.5±0.02c
	Glycerate	20.6±0.64a	26.3±2.16b	24.8±0.72b	24.6±1.61b
	Gluconate	8.1±0.2c	6.4±0.4b	9.6±0.2d	5.7±0.4a
Phosphorylated compounds	Glucose-6-P	6.2±0.5c	2.5±0.3b	7.2±0.7d	0.9±0.5a
	Fructose-6-P	3.21±0.08c	1.56±0.24b	4.08±0.21d	0.41±0.23a
	Ribose-5-P	0.230±0.010b	0.279±0.007c	0.278±0.027c	0.213±0.011a
	Ribulose-5-P	0.125±0.008c	0.094±0.035b	0.154±0.024d	0.045±0.007a
	Sedoheptulose-7-P	0.74±0.12c	0.29±0.03b	1.00±0.19d	0.05±0.03a
	Glycerol-3-P	8.2±1.0b	3.1±0.2a	8.7±1.2b	1.9±0.6a
	Glycerate-2-P	1.09±0.17b	0.62±0.06a	1.34±0.23c	0.72±0.06a
	Glycerate-3-P	4.9±1.2c	4.0±0.2bc	3.0±0.8b	1.4±1.1a
	Gluconate-6-P	0.33±0.01ab	0.38±0.04b	0.29±0.01a	0.52±0.07c
	Inositol-1-P	1.53±0.05ab	1.96±0.12c	1.64±1.31b	1.37±0.68a
Amino acids	Alanine	30.4±5.56c	18.2±2.14a	24.6±2.27b	16.5±1.23a
	Valine	7.6±0.4ab	6.5±0.6a	7.2±1.2ab	8.4±0.5b
	Leucine	0.45±0.14a	0.82±0.03b	0.67±0.14b	2.72±0.53c
	Isoleucine	5.56±0.86c	4.15±0.12a	4.67±0.03ab	4.82±0.085b
	Proline	11.1±4.32a	40.8±5.69b	14.1±5.12a	34.5±3.23b
	Glycine	7.8±0.3bc	8.3±0.1c	6.6±0.6a	6.0±0.6a
	Norleucine	0.6±0.3a	4.4±2.0b	0.8±0.6a	0.6±0.2a
	Serine	33.0±0.95b	53.1±4.46d	23.6±6.00a	45.7±2.78c
	Threonine	4.49±0.03a	5.17±0.24b	4.31±0.29a	4.91±0.03b
	S-Methylcysteine	0.025±0.004a	0.029±0.001a	0.070±0.019bc	0.064±0.001b
	ß-Alanine	0.69±0.01b	0.69±0.03ab	0.73±0.01c	0.67±0.02a
	Homoserine	0.93±0.06d	0.25±0.03a	0.35±0.07b	0.47±0.07c

	Methionine	0.32±0.01a	0.42±0.07b	0.61±0.04c	0.63±0.01c
	Cysteine	0.20±0.06a	0.25±0.04a	0.28±0.06b	0.22±0.03ab
	L,L-Cystathionine	0.061±0.011c	0.044±0.004b	0.039±0.001b	0.021±0.006a
	Homocysteine	0.178±0.003a	0.322±0.008b	0.271±0.040b	0.302±0.027b
	Phenylalanine	0.89±0.17a	0.56±0.24a	1.35±0.09b	1.44±0.08b
	Arginine	0.041±0.011a	0.031±0.009a	0.134±0.011b	0.040±0.028b
	Histidine	0.30±0.03a	0.30±0.03ab	0.33±0.01b	0.33±0.02ab
	Lysine	0.7±0.1a	0.7±0.3a	4.8±0.1b	6.8±1.0c
	Tyrosine	2.7±0.1c	0.4±0.6a	2.1±0.2b	1.8±0.4b
	Tryptophan	0.11±0.05a	0.11±0.1a	0.15±0.07a	0.16±0.11a
	Glutamine	11.1±1.72a	10.2±3.00a	9.02±4.06a	9.31±3.36a
	Glutamate	57.9±4.62c	64.9±5.04cd	39.3±5.13a	46.2±4.22b
	Ornithine	0.24±0.01b	0.20±0.03a	0.49±0.04d	0.41±0.01c
	γ-Aminobutyricacid (GABA)	30.6±0.54a	55.8±10.1b	82.9±7.09c	78.8±17.5c
Organic acids	Pyruvate	0.52±0.04b	0.73±0.08c	0.42±0.11a	0.47±0.01a
	Lactate	0.32±0.02b	0.19±0.02a	0.42±0.01c	0.75±0.04d
	Glycolate	0.63±0.02a	1.24±0.20c	0.98±0.03b	1.15±0.14bc
	Succinate	3.6±0.2b	2.4±0.7b	2.6±0.3b	0.5±0.1a
	Fumarate	2.9±0.4b	3.1±0.4b	3.3±0.1b	1.8±0.4a
	Malate	24.7±2.12a	32.4±2.31b	31.4±1.96ab	30.0±3.18ab
	Pyroglutamate	81.1±2.66c	77.3±4.44bc	54.5±9.48a	69.5±11.7b
	L-Aspartate	104.4±5.930c	91.73±6.042b	113.6±5.668d	63.49±9.88a
	Citrate	15.6±0.88b	14.3±2.20b	19.5±1.38c	10.8±2.19a
	Isocitrate	1.8±0.4b	1.6±0.1b	1.3±0.3b	0.9±0.2a
	cis-Aconitate	0.24±0.02b	0.20±0.02b	0.37±0.01c	0.15±0.01a
	2-Isopropylmalate	0.149±0.012b	0.115±0.01b	0.111±0.008b	0.061±0.004a
	α-Ketoglutarate	0.75±0.18b	0.81±0.02b	0.50±0.03a	0.61±0.01a
	2-Hydroxyglutarate	0.326±0.004b	0.428±0.087bc	0.270±0.011a	0.169±0.022a
	Phosphoenolpyruvate	0.300±0.065c	0.039±0.004a	0.155±0.007b	0.023±0.014a
	Oxalic acid	9.85±0.87ab	11.2±1.12b	10.7±0.69b	8.56±0.81a
	Gluconate-1,5-lactone	1.3±0.2a	1.9±0.1b	1.0±0.5ab	2.9±0.4c
	Pantothenic acid	0.267±0.001a	0.210±0.010b	0.269±0.061a	0.161±0.059a
	Threonic acid	9.51±0.93a	8.74±3.17a	19.0±0.01b	9.56±0.01a
	Threonic acid-1,4-lactone	0.55±0.05a	1.01±0.13b	1.49±0.05c	0.56±0.05a
Miscellaneous	Adenine	4.24±0.20a	5.76±0.23b	20.5±0.14c	23.0±0.02d
	Adenosine	0.51±0.02b	0.29±0.02a	0.28±0.08a	0.59±0.06b
	Guanine	0.212±0.005b	0.283±0.01a	0.316±0.060c	0.340±0.018d
	Thymine	0.113±0.008a	0.366±0.044c	0.112±0.012a	0.175±0.015b
	Uracil	0.22±0.01a	0.28±0.03b	0.32±0.01b	0.30±0.01b
	cytosine	0.012±0.001a	0.748±0.045c	0.075±0.032b	0.069±0.019b
	Putrescine	0.78±0.09a	1.14±0.09b	0.82±0.04a	0.71±0.81a
	Shikimate	0.62±0.11bc	0.49±0.08b	0.48±0.02b	0.33±0.2ab
	Dopamine	36.2±6.88c	24.3±0.46ab	28.1±0.04b	23.4±0.06a

Supplementary Table S3: Metabolites changes in different fractions of sugarbeet. Data are means ±SD of n=6 experiments. Data groups of significant difference were assessed with Fisher's LSD ($p<0.05$) and labelled with different letters.

	Metabolites	Extra chloroplast space [r.u./µg chlorophyll]				Chloroplast [r.u./µg chlorophyll]			
		**Control 3h**	**Stress 3h**	**Control 339h**	**Stress 339h**	**Control 3h**	**Stress 3h**	**Control 339h**	**Stress 339h**
Sugars	Glucose	5.8±0.9g	5.4±0.9f	3.9±0.6d	4.7±0.9e	0.7±0.2a	1.0±0.1b	1.0±0.1ab	1.3±0.1c
	Fructose	3.3±0.9f	2.9±0.4e	2.3±0.4d	2.1±0.4d	0.4±0.2a	0.6±0.1b	0.7±0.2b	0.9±0.2c
	Galactose	0.41±0.07c	0.60±0.11d	0.97±0.24e	1.00±0.24e	0.05±0.02a	0.29±0.09bc	0.24±0.08b	0.24±0.09b
	Lactose	0.013±0.009c	0.029±0.005d	0.01±0.006c	0.012±0.002c	0.003±0.002a	0.004±0.002a	0.006±0.005b	0.007±0.004b
	Sucrose	11.7±1.41d	13.3±1.51e	5.45±1.66c	6.66±0.89c	3.04±0.56b	3.48±0.82b	2.98±1.13b	1.13±0.41a
	Cellobiose	0.0232±0.0053e	0.0099±0.0003d	0.0134±0.0033d	0.0182±0.0020e	0.0032±0.0011b	0.0077±0.0007c	0.0002±0.0005a	0.0029±0.0003b
	Maltose	0.0049±0.0010b	0.0036±0.0011b	0.0046±0.0013b	0.0066±0.0011c	0.0033±0.0011b	0.0035±0.0012b	0.0012±0.0003a	0.0009±0.0009a
	Trehalose	0.032±0.003e	0.040±0.006f	0.018±0.005d	0.023±0.004d	0.001±0.002a	0.011±0.005c	0.002±0.003a	0.006±0.002b
	Melibiose	0.045±0.008bc	0.060±0.009c	0.045±0.008bc	0.041±0.011b	0.013±0.003a	0.043±0.015b	0.013±0.012a	0.050±0.009b
	Arabinose	0.45±0.12c	0.80±0.14d	0.37±0.15c	1.32±0.40e	0.03±0.02a	0.20±0.06b	0.17±0.12b	0.45±0.10c
	Ribose	1.05±0.37c	1.82±0.15d	1.27±0.36d	3.72±1.46e	0.02±0.04a	0.32±0.12ab	0.61±0.27b	0.21±0.13a
	Rhamnose	0.011±0.001e	0.013±0.001e	0.009±0.001d	0.020±0.004f	0.002±0.001a	0.004±0.002b	0.007±0.001c	0.004±0.001b
	Glycerate	1.9±0.3d	1.8±0.4d	1.5±0.2c	2.1±0.3d	0.5±0.1a	1.4±0.4c	0.5±0.1a	0.7±0.2b
	Gluconate	0.900±0.037f	0.545±0.019d	0.650±0.022de	0.515±0.183d	0.008±0.009a	0.133±0.037c	0.100±0.029b	0.079±0.017b
	Xylose	0.099±0.023e	0.170±0.012f	0.054±0.010c	0.068±0.002d	0.025±0.001b	0.025±0.007b	0.010±0.005a	0.023±0.01b
	Ribitol	1.5±0.2e	1.6±0.2f	1.02±0.3d	1.5±0.2e	0.3±0.1a	0.4±0.1b	0.3±0.1a	0.5±0.1c
	Mannitol	0.012±0.002c	0.022±0.006d	0.008±0.002c	0.030±0.005d	0.001±0.001a	0.002±0.001ab	0.001±0.001a	0.004±0.001b
	Inositol	0.7±0.2c	1.7±0.5d	0.8±0.2c	1.7±0.5d	0.3±0.1a	0.4±0.1ab	0.3±0.08a	0.3±0.07ab
Phosphorylated compounds	Glucose-6-P	0.45±0.10f	0.20±0.04c	0.35±0.07def	0.07±0.04b	0.29±0.04de	0.11±0.01c	0.36±0.04f	0.07±0.02a
	Fructose-6-P	0.204±0.069ef	0.123±0.049d	0.185±0.062def	0.032±0.022b	0.175±0.055de	0.060±0.008c	0.223±0.073f	0.012±0.005a
	Ribose-5-P	0.009±0.003c	0.007±0.005ab	0.010±0.004c	0.004±0.001a	0.017±0.002d	0.034±0.009e	0.016±0.001d	0.010±0.001bc
	Ribulose-5-P	0.005±0.001a	0.002±0.002a	0.005±0.001a	0.002±0.001a	0.011±0.002b	0.020±0.001c	0.013±0.001b	0.010±0.002ab

	Seduheptulose-7-P	0.0002±0.0029a	0.0086±0.0036ab	0.0132±0.0030ab	0.0009±0.0012a	0.1408±0.0444d	0.0428±0.0103c	0.1318±0.0442d	0.0094±0.0014b
	Glycerol-3-P	0.54±0.2e	0.20±0.06b	0.40±0.16cd	0.02±0.01a	0.48±0.16de	0.30±0.12c	0.41±0.14de	0.31±0.14c
	Glycerate-2-P	0.067±0.015cd	0.040±0.016bc	0.074±0.016cde	0.051±0.012bcd	0.04±0.003bc	0.040±0.005b	0.049±0.001c	0.026±0.004a
	Glycerate-3-P	0.09±0.06b	0.27±0.05c	0.08±0.03ab	0.05±0.07a	0.76±0.11d	0.22±0.15c	0.55±0.18d	0.10±0.02b
	Gluconate-6-P	0.015±0.009a	0.015±0.010a	0.012±0.010a	0.025±0.006a	0.042±0.022ab	0.116±0.01c	0.015±0.01a	0.076±0.011b
	Inositol-1-P	0.122±0.019d	0.20±0.008e	0.091±0.024bc	0.108±0.038bcd	0.058±0.011a	0.090±0.009bc	0.062±0.015a	0.102±0.033cd
Amino acids	Alanine	2.0±1.4c	1.1±0.3ab	1.5±0.4b	1.3±0.2b	1.1±0.3ab	1.4±0.3b	0.5±0.1a	0.5±0.1a
	Valine	0.64±0.13e	0.53±0.13d	0.54±0.12d	0.75±0.10e	0.42±0.03b	0.31±0.08c	0.15±0.05a	0.26±0.06bc
	Leucine	0.045±0.018b	0.062±0.049cd	0.050±0.054c	0.234±0.045e	0.028±0.022b	0.032±0.031b	0.010±0.004a	0.062±0.051d
	Isoleucine	0.31±0.08d	0.30±0.13d	0.27±0.06cd	0.36±0.14e	0.23±0.04bc	0.23±0.02bc	0.14±0.01a	0.21±0.04b
	Proline	0.7±0.2b	3.4±1.2c	0.8±0.3b	3.1±1.4c	0.6±0.3ab	1.0±0.3b	0.4±0.1a	0.4±0.1a
	Glycine	0.71±0.15de	0.64±0.12d	0.43±0.12d	0.50±0.14d	0.14±0.04a	0.30±0.04c	0.13±0.04a	0.20±0.06b
	Norleucine	0.039±0.009bc	0.020±0.016ab	0.046±0.004c	0.047±0.01c	0.019±0.003ab	0.015±0.004a	0.007±0.001a	0.011±0.003a
	Serine	2.7±0.8d	4.5±1.5e	1.3±0.5c	4.1±1.3e	1.2±0.4bc	1.3±0.4c	0.5±0.1a	0.8±0.2ab
	Threonine	0.35±0.12e	0.41±0.14f	0.25±0.09d	0.40±0.14f	0.18±0.06c	0.18±0.06c	0.11±0.03a	0.20±0.05b
	S-Methylcysteine	0.0015±0.0011bc	0.0059±0.0015e	0.0021±0.0014bc	0.0054±0.0019e	0.0011±0.0012ab	0.0034±0.0023d	0.0008±0.0011a	0.0016±0.0022ab
	ß-Alanine	0.064±0.004e	0.055±0.019e	0.045±0.011d	0.060±0.011e	0.016±0.007b	0.021±0.007c	0.017±0.002b	0.014±0.003a
	Homoserine	0.074±0.015c	0.094±0.007d	0.062±0.015bc	0.090±0.008d	0.035±0.005bc	0.043±0.002b	0.010±0.002a	0.021±0.004ab
	Methionine	0.033±0.005d	0.037±0.008de	0.043±0.009e	0.054±0.004f	0.011±0.003b	0.022±0.006c	0.002±0.0003a	0.013±0.003b
	Cysteine	0.014±0.004b	0.022±0.004c	0.012±0.001b	0.018±0.006c	0.014±0.001bc	0.016±0.001c	0.010±0.001a	0.014±0.001bc
	L,L-Cystathionine	0.0040±0.0025d	0.0036±0.0032c	0.0024±0.0013b	0.0008±0.0002a	0.0006±0.0013a	0.0012±0.0010a	0.0010±0.0009a	0.0019±0.0006a
	Homocysteine	0.014±0.002c	0.025±0.002e	0.016±0.002d	0.019±0.004d	0.007±0.002b	0.012±0.002c	0.003±0.001a	0.020±0.005d
	Phenylalanine	0.07±0.02e	0.03±0.02b	0.08±0.01f	0.12±0.01g	0.03±0.01a	0.04±0.01c	0.03±0.01a	0.05±0.01d
	Arginine	0.0001±0.0001a	0.0003±0.0002a	0.0074±0.0050bc	0.0079±0.0013c	0.0079±0.0018bc	0.0073±0.0016b	0.0045±0.0011ab	0.0120±0.0027d
	Histidine	0.024±0.001c	0.032±0.004d	0.022±0.003b	0.027±0.006cd	0.003±0.001a	0.003±0.001a	0.005±0.001a	0.009±0.004a
	Lysine	0.24±0.04d	0.30±0.01e	0.29±0.06e	0.53±0.15f	0.03±0.01a	0.06±0.01b	0.09±0.02b	0.12±0.01c
	Tyrosine	0.240±0.081d	0.201±0.002e	0.116±0.030bc	0.087±0.05bc	0.066±0.024ab	0.096±0.037bc	0.046±0.018a	0.104±0.037bc

	Tryptophan	0.011±0.005c	0.011±0.002c	0.009±0.003bc	0.021±0.002bc	0.001±0.001a	0.004±0.001b	0.001±0.001a	0.005±0.002b
	Glutamine	0.46±0.17b	0.21±0.06a	0.60±0.04b	0.62±0.1b	1.06±0.36c	1.76±0.41d	0.23±0.17a	0.63±0.27b
	Glutamate	2.6±0.9bcd	3.2±0.6cde	1.3±0.4a	3.9±0.4de	3.3±0.3cd	4.1±0.4e	2.4±0.1b	1.3±0.6a
	Ornithine	0.0140±0.0024c	0.0010±0.0005a	0.0294±0.0045d	0.030±0.0033d	0.0154±0.0064c	0.0389±0.0090e	0.0114±0.0017b	0.0220±0.0103c
	γ-Aminobutyricacid (GABA)	3.25±1.12cd	4.20±1.16d	5.20±1.94de	8.81±1.03f	0.18±0.06a	2.47±0.9bc	1.88±0.86b	1.91±0.91b
Organic acids	Pyruvate	0.051±0.011c	0.070±0.012d	0.038±0.003b	0.026±0.015b	0.006±0.005a	0.002±0.005a	0.006±0.004a	0.003±0.004a
	Lactate	0.025±0.008b	0.009±0.009a	0.034±0.012c	0.070±0.003d	0.013±0.003a	0.020±0.004b	0.013±0.003a	0.011±0.011a
	Glycolate	0.062±0.010bc	0.089±0.018d	0.064±0.010bc	0.098±0.004d	0.015±0.008a	0.076±0.02c	0.016±0.01a	0.036±0.01b
	Succinate	0.382±0.045c	0.498±0.054d	0.157±0.023b	0.040±0.030a	0.032±0.013a	0.052±0.014a	0.061±0.016a	0.013±0.005a
	Fumarate	0.28±0.04c	0.29±0.01c	0.21±0.06b	0.22±0.07b	0.01±0.01a	0.05±0.01a	0.04±0.02a	0.03±0.01a
	Malate	2.42±0.88cd	3.61±0.71de	2.12±0.14c	3.05±0.49d	0.01±0.011a	0.2±0.0843b	0.05±0.06a	0.2±0.0971b
	Pyroglutamate	7.0±1.2e	6.0±0.7d	5.4±1.1c	7.8±0.1de	2.1±0.7ab	2.5±0.7b	1.9±0.7ab	1.1±0.3a
	L-Aspartate	8.4±3.0e	7.9±2.6d	7.4±2.1d	5.6±1.8c	1.9±0.9b	1.5±0.6ab	1.6±0.7ab	1.1±0.5a
	Citrate	1.73±0.53c	2.68±0.20d	1.29±0.43bc	2.38±0.10d	0.03±0.01a	0.26±0.07b	0.24±0.1b	0.11±0.05b
	Isocitrate	0.133±0.074e	0.119±0.032e	0.089±0.024de	0.106±0.021d	0.002±0.001a	0.033±0.018c	0.006±0.001ab	0.017±0.007ab
	cis-Aconitate	0.0263±0.0060f	0.0182±0.0041d	0.0242±0.0061e	0.0130±0.0045c	0.0005±0.0003a	0.0022±0.0014ab	0.0045±0.0022b	0.0021±0.0010ab
	2-Isopropylmalate	0.011±0.003g	0.008±0.001f	0.006±0.001ef	0.004±0.001bc	0.006±0.002de	0.005±0.003cd	0.002±0.001a	0.003±0.001ab
	α-Ketoglutarate	0.041±0.028d	0.042±0.002d	0.036±0.008d	0.058±0.013e	0.005±0.002b	0.012±0.005c	0.001±0.001a	0.005±0.001b
	2-Hydroxyglutarate	0.033±0.008e	0.032±0.011e	0.015±0.002c	0.020±0.005d	0.003±0.002a	0.020±0.009d	0.008±0.004b	0.003±0.001a
	Phosphoenolpyruvate	0.0135±0.0032c	0.0039±0.0012b	0.0113±0.0032c	0.0031±0.0018b	0.0017±0.0003a	0.0028±0.0001ab	0.0003±0.0001a	0.0005±0.0002a
	Oxalic acid	0.77±0.33d	1.00±0.27e	0.70±0.21d	0.54±0.19cd	0.19±0.06a	0.31±0.1b	0.18±0.06a	0.23±0.08ab
	Gluconate-1,5-lactone	0.080±0.009c	0.112±0.021d	0.044±0.025abc	0.073±0.019bcd	0.015±0.008a	0.022±0.002ab	0.016±0.008a	0.022±0.008a
	Pantothenic acid	0.024±0.008d	0.014±0.009c	0.016±0.01c	0.011±0.003c	0.006±0.003a	0.011±0.004c	0.008±0.003b	0.008±0.006ab
	Threonic acid	0.6±0.2bc	0.7±0.04c	1.1±0.4d	0.7±0.2c	0.5±0.1b	0.5±0.2b	0.5±0.1b	0.3±0.1a
	Threonic acid-1,4-lactone	0.03±0.01a	0.09±0.01d	0.04±0.02b	0.02±0.01a	0.04±0.02bc	0.02±0.01a	0.13±0.06e	0.05±0.01c

Miscellaneous	Adenine	0.55±0.15d	0.44±0.15cd	1.20±0.42e	2.12±0.38f	0.03±0.01a	0.21±0.05b	0.54±0.2d	0.32±0.13c
	Adenosine	0.048±0.012f	0.004±0.003a	0.015±0.005c	0.040±0.003e	0.012±0.007bc	0.053±0.016g	0.009±0.002b	0.030±0.008d
	Guanine	0.019±0.002e	0.011±0.009d	0.021±0.002f	0.031±0.004g	0.005±0.001b	0.007±0.002c	0.003±0.001a	0.007±0.002c
	Thymine	0.0122±0.0026c	0.0349±0.0060e	0.0080±0.0062b	0.0164±0.0042d	0.0007±0.0021a	0.0004±0.0023a	0.0006±0.0022a	0.0003±0.0019a
	Uracil	0.0185±0.0031b	0.0228±0.0018c	0.0245±0.0037c	0.0267±0.0075d	0.0067±0.0023a	0.0068±0.0021a	0.0069±0.0020a	0.0056±0.0019a
	cytosine	0.0012±0.0052a	0.0730±0.0235c	0.0049±0.0054b	0.0058±0.0054b	0.0002±0.0012a	0.0008±0.0014a	0.0011±0.0013a	0.0002±0.0016a
	Putrescine	0.058±0.006c	0.094±0.020e	0.043±0.006b	0.085±0.007d	0.018±0.004a	0.031±0.001b	0.031±0.010b	0.032±0.0061b
	Shikimate	0.042±0.028c	0.041±0.018c	0.036±0.011c	0.030±0.015bc	0.006±0.002a	0.031±0.011c	0.013±0.012a	0.020±0.005b
	Dopamine	2.6525±.1793c	2.2881±0.5620b	2.8502±0.2321b	2.4406±0.3405b	0.0001±0.0001a	0.0004±.0005a	0.0001±0.0000a	0.0004±0.0006a
